# Mitophagy Dysfunction in Alzheimer's Disease

**DOI:** 10.1002/alz70855_106610

**Published:** 2025-12-24

**Authors:** Maggie N Benson, Keith P Smith, Vivien Csikos, Heather M Wilkins

**Affiliations:** ^1^ University of Kansas Medical Center, Kansas City, KS, USA; ^2^ 3901 rainbow blvd, Kansas City, KS, USA; ^3^ University of Kansas Alzheimer's Disease Research Center, Kansas City, KS, USA; ^4^ University of Kansas Alzheimer's Disease Research Center, Fairway, KS, USA; ^5^ Department of Neurology, University of Kansas School of Medicine, Kansas City, KS, USA

## Abstract

**Background:**

Alzheimer's disease (AD) is a common neurodegenerative disorder marked by amyloid beta (Aβ) plaques and neurofibrillary tangles. Studies have revealed that damaged mitochondria accumulate across various AD disease models, suggesting disrupted mitochondrial quality control pathways. Mitophagy—the cellular process that removes dysfunctional mitochondria—has been shown to be impaired in AD, though its exact relationship to disease mechanisms remains unclear. This study investigates the relationship between mitophagy mechanisms and AD pathophysiology.

**Methods:**

Whole brain from 5xFAD and wild‐type (WT) mice was use to collect whole cell, mitochondrial, and autophagosome components (AP). Mitochondrial DNA (mtDNA) copy number was measured from whole brain and AP fractions using qPCR. iPSC‐derived cerebral organoid models were generated from both non‐AD and sporadic AD (sAD) sources. These models were then separated into AP fractions and mtDNA copy number was measured using qPCR. Postmortem human brain was fractionated to collect whole cell, mitochondrial, and AP fractions from non‐demented (ND) and sAD subjects. Aβ levels were measured in fractions using ELISA kits. iPSCs where used to derive neurons from ND and sAD subjects and lysosome number and autophagosome events were measured using LysoTracker and DAPRed fluorescent dyes.

**Results:**

We observed a significant reduction in AP mtDNA content from 5xFAD mice from 2 months of age, while whole brain mtDNA was elevated in 5xFAD mice at 2 months of age but reduced at 12 months of age. Organoids derived from sAD iPSC donors also had reduced AP mtDNA content. Aβ levels were increased in whole and AP fractions in 5xFAD mouse samples, cerebral organoid models, and human postmortem brain. iPSC derived neurons from sAD donors had reduced lysosome content and autophagy events.

**Conclusions:**

Overall mitophagy is impaired across mouse and iPSC models of AD. Associations with Aβ pathology and other underlying mechanisms requires further investigation.

